# Viral Infection and Connexin Dysfunction in the Heart

**DOI:** 10.1007/s11886-025-02227-6

**Published:** 2025-03-27

**Authors:** Chelsea M. Phillips, James W. Smyth

**Affiliations:** 1 Fralin Biomedical Research Institute at Virginia Tech Carilion, Roanoke, VA 24016 USA; 2 Department of Biological Sciences, College of Science, Virginia Tech, Blacksburg, VA 24061 USA; 3 Department of Basic Science Education, Virginia Tech Carilion School of Medicine, Roanoke, VA 24016 USA; 4 Center for Vascular and Heart Research, Fralin Biomedical Research Institute at Virginia Tech Carilion, Roanoke, VA 24016 USA; 5 Department of Biomedical Engineering and Mechanics, College of Engineering, Virginia Tech, Blacksburg, VA 24061 USA

**Keywords:** Connexin, Gap Junction, Virus, Myocarditis, Infection, Arrhythmia

## Abstract

**Purpose of review:**

Gap junctions, comprising connexin proteins, enable the direct intercellular electrical coupling of cardiomyocytes, and disruption of this process is arrhythmogenic. In addition, gap junctions effect metabolic coupling and of relevance to this review, propagate host antiviral immune responses. Accordingly, connexins have emerged as viral targets during infection. This review summarizes current knowledge regarding contributions of inflammation *vs* virally encoded factors in driving alterations to cardiac gap junction function.

**Recent findings:**

In addition to host immune-mediated effects on cardiac electrophysiology and gap junctions in myocarditis, there is now increasing appreciation for virally encoded factors targeting connexin function in acute/active infection.

**Summary:**

We now know diverse viral species have independently evolved to directly target connexin function during infection. Understanding both the direct and indirect effects of viral infection on cardiac gap junctions is critical to inform treatment strategies and development of novel therapeutics for acute infection as a distinct disease process from chronic myocarditis.

## Introduction

### Connexins

Gap junctions, comprising connexin proteins, allow for the direct exchange of ions and small molecules between adjacent cells. Six connexins oligomerize to form a connexon hemichannel, which is trafficked to the plasma membrane. On the cell surface, connexin hemichannels can facilitate communication between extracellular and intracellular environments while two connexons on apposing cells can dock to form a gap junction channel. Gap junction channels coalesce into dense arrays coupling the cytoplasms of adjacent cells to effect direct electrical and metabolic intercellular communication [1, 2]. Connexins are tetraspan membrane proteins, possessing intracellular N- and C-termini, two extracellular loops, and one intracellular loop [3, 4]. Many amino acid residues occurring the connexin C-termini are subject to posttranslational modifications (PTMs) which dynamically regulate connexin function and localization [5]. The surprisingly short half-life of connexins renders such modifications extremely potent in rapid modulation of gap junction function and intercellular coupling [6, 7].

Of the 21 human connexins, connexin43 (Cx43, gene name *GJA1*) is the most ubiquitously expressed and is important for action potential propagation in the working myocardium, resulting in coordinated cardiac contraction [8–10]. Aside from PTMs, mechanisms regulating Cx43 expression and function include transcription and translation. Internal translation of *GJA1*, leading to the expression of a 20 kilodalton (kDa) isoform of Cx43 (GJA1-20k), is a critical regulatory mechanism [11]. GJA1-20k acts as an auxiliary chaperone protein necessary for Cx43 forward trafficking, as loss of GJA1-20k expression reduces gap junction plaques at cell–cell borders in vitro and the intercalated disc (ID) in cardiomyocytes in vivo [11–14]. Furthermore, transcriptional regulation of *GJA1* has been demonstrated to influence its translation. Variability within the 5’ untranslated region (UTR) of *GJA1* dictates translation initiation: the various 5’ UTR *GJA1* isoforms are either permissive or inhibitory to internal translation, or GJA1-20k expression, with its expression ultimately influencing gap junction formation [13]. Viral hijacking of cellular translation machinery, and bypass of cap-dependent translation initiation, are well described and therefore may be of high relevance to modulation of gap junctions during cardiac infection given the central role of translation in heart disease progression [15, 16]. Altogether, this highlights the multifaceted nature of Cx43 regulation and its importance in cardiac function.

Other connexins expressed by the various cell types comprising the mammalian heart include Cx40, Cx45, and Cx37, with mouse cardiac tissue also expressing Cx30.2 [9, 17–20]. As connexins are essential for the coordinated contraction of the working myocardium, unsurprisingly, aberrancies in connexin expression, function, or localization are common across various cardiovascular diseases, including viral infection of the heart [21–24].

### Connexins and Immune Function: Innate and Adaptive Immune Response

Gap junctions uniquely contribute to the cell intrinsic antiviral immune response through facilitating propagation of innate and adaptive immune signals. This posits gap junctions as logical targets for viruses, where preventing GJIC dampens the cell intrinsic antiviral immune response and would therefore promote viral replication and propagation. Of relevance, the cell-intrinsic innate immune signaling molecule cyclic guanosine monophosphate-adenosine monophosphate (cGAMP) is synthesized by cGAMP synthase (cGAS) upon detection of exogenous nucleic acids in the cytosol and can traverse gap junctions between infected cells and their uninfected neighbors [25, 26]. cGAMP activates the receptor stimulator of interferon genes (STING), which induces STING translocation from the endoplasmic reticulum (ER) to the perinuclear region and leads TANK-binding kinase 1 (TBK1)-mediated phosphorylation of both STING and interferon regulatory factor 3 (IRF3) [27–29]. This phosphorylation event leads to IRF3 dimerization, translocation to the nucleus, and production of type I interferon (IFN) through binding to interferon-stimulated response element (ISRE) sequences [30, 31]. As mentioned above, cGAMP has been demonstrated to spread between cells via GJIC, therefore precipitating activation of the IFN response in uninfected, bystander cells [32, 33]. In addition to its role in innate immune signaling, GJIC also contributes to adaptive immune responses. Specifically, short linear peptides, such as viral antigens, can be exchanged through gap junctions from an infected cell to uninfected neighboring cells and presumably, immune cells. This allows uninfected cells to present the viral antigens via their major histocompatibility complexes, allowing for cytotoxic T lymphocyte-mediated cell killing and effectively creating a ‘firewall’ within infected tissues to limit viral spread [34].

### Connexins, Metabolic Coupling, and Cytotoxicity

Conversely, gap junction function can also induce cell damage. Specifically, inducing apoptosis within a given cell population increases bystander cell vulnerability to apoptotic triggers, which occurs in a gap junction-dependent manner [35, 36]. Key signaling mediators predisposing bystander cells to undergo apoptosis include inositol triphosphate (IP_3_) and intracellular Ca^2+(36, 37)^. Furthermore, Cx43 hemichannel activity has been demonstrated to propagate cell death beyond regions of direct coupling, with intracellular Ca^2+^ identified as a key signaling molecule mediating this effect [37]. Within the context of the heart, pharmacological inhibition of both plasma membrane and mitochondrial Cx43 hemichannels reduced the infarct size in an ex vivo Langendorff model of ischemia–reperfusion [38]. Moreover, Cx43-heterozygous mice subjected to left anterior descending coronary artery ligation had a significantly smaller infarct size compared to wild-type (WT) mice [39]. In a mouse model of acute ischemia, expression of C-terminally truncated Cx43, a Cx43 construct preventing pH-mediated closure of gap junctions, increases cardiac infarct size, demonstrating gap junction activity during ischemia can further propagate myocardial injury [40, 41]. With gap junctions and hemichannels at the intersection of the cell intrinsic immune response and cell injury and death, it is unsurprising that viral infection commonly results in connexin dysfunction.

### Cardiac Connexins and Inflammation

Viral infection is the leading cause of acute myocarditis, a disease characterized by aberrant inflammation that ultimately damages the myocardium [42–44]. Research investigating the immune response in the context of acute myocarditis has focused primarily on understanding innate immune activation, particularly inflammasome formation and pro-inflammatory cytokine levels. In cardiac tissue from acute myocarditis patients, upregulation of inflammasome components has been detected in cardiomyocytes, fibroblasts, endothelial cells, and leukocytes [45]. Accordingly, an in vivo model of Coxsackie virus B3 (CVB3)-induced myocarditis demonstrated a significant increase in the inflammasome components caspase-1 and apoptosis-associated speck-like protein containing a caspase-1 recruiting domain (ASC). Accordingly, treatment with the caspase-1 inhibitor Ac-YVAD-CHO increased survival and prevented cardiac damage [46]. As cardiac viral titers in caspase-1 inhibitor-treated mice were comparable to those of untreated mice, this confirmed a key role for inflammasome activation in the progression of acute viral myocarditis [46]. Patients with acute myocarditis also have increased serum levels of pro-inflammatory cytokines, including interleukin (IL)−1ɑ, IL-1β, and tumor necrosis factor (TNF)-ɑ [47]. Upregulation of pro-inflammatory cytokines, such as IL-1β, TNF-ɑ, and IFN-ɣ, has also been demonstrated in vivo in several mouse models of acute viral myocarditis [46, 48, 49]. Moreover, these in vivo models identify expression of such pro-inflammatory cytokines as causative in viral myocarditis progression [46, 50]. Of clinical relevance, treatment with IL-1β neutralizing antibody increased survival and decreased the myocarditis score in CVB3-induced mouse model of myocarditis [46]. Likewise, IL-1β mRNA levels positively correlated with cardiac hypertrophy and fibrosis in a post-myocarditis dilated cardiomyopathy mouse model, indicating the potential relationship between IL-1β expression and the progression of viral myocarditis to dilated cardiomyopathy [49].

With inflammation identified as a key contributor to acute myocarditis, the negative impact of pro-inflammatory cytokines on cardiomyocyte function is unsurprising. Two well-described cytokines involved in the progression of myocarditis, TNF-ɑ and IL-1β, have been demonstrated to impair cardiomyocyte ion currents, compromise contractile function, and disrupt Ca^2+^ homeostasis [51–55]. Moreover, inflammation has also been demonstrated to influence connexin expression, localization, and PTMs [48, 56–58]. Inducing inflammation via lipopolysaccharide (LPS) treatment or regional hepatic ischemia/reperfusion injury decreases cardiac transcript levels of *Gja1* mRNA [56]. In particular, TNF-ɑ and IL-1β have been demonstrated to alter connexin regulation. Treatment with TNF-ɑ decreased *Gja1* promoter activity in vitro, and cardiac TNF-ɑ overexpression in vivo disrupted Cx43 localization, reducing levels at the ID [56, 57]. Meanwhile, IL-1β has been demonstrated to modulate Cx43 PTMs, including Cx43 serine368 (Cx43-S368) hyperphosphorylation in vitro and in vivo, a post-translational modification that decreases channel open probability and gap junction function [58–61]. Within the context of CVB3-induced myocarditis, blunting expression of TNF-ɑ and IFN-ɣ in vivo via treatment with atorvastatin, a 3-hydroxy-3-methylglutaryl co-enzyme A (HMG-CoA) inhibitor, rescued cardiac expression and localization of Cx43 and Cx45 [48]. This highlights inflammation as a driver of connexin dysfunction in CVB3-induced myocarditis. Together, these studies support a significant contribution from inflammation during acute viral myocarditis in influencing connexin dynamics, and therefore gap junction function.

## Cardiac Connexins and Viral Infection

### Known Etiological Agents of Viral Myocarditis

#### Adenovirus

Adenoviral infection has been identified as the leading etiological cause of viral myocarditis across all age groups, with viral genomes detected within cardiac tissue biopsied from both suspected and confirmed viral myocarditis cases [62–64]. Human adenovirus (HAdV) types 2 and 5 are primarily associated with viral myocarditis; however, incidences of viral myocarditis and sudden cardiac death due to other types of HAdV, such as HAdV-3, have also been reported [62, 65, 66]. Furthermore, HAdV-2 has also been detected in myocardial biopsies from patients with idiopathic left ventricular dysfunction, revealing the widespread role of adenoviruses across various cardiovascular complications [66]. This indicates the importance in understanding molecular mechanisms, such as cardiac connexin dysfunction, that contribute to the progression of adenoviral myocarditis.

Identification of the cardiotropic mouse adenovirus type 3 (MAdV-3) has aided in the establishment of an in vivo model of adenoviral myocarditis, allowing for investigation of molecular changes occurring in the acute phase of adenoviral myocarditis [67, 68]. Acute MAdV-3 infection resulted in an arrhythmogenic substrate, as indicated by decreased conduction velocity and increased action potential duration, which preceded appreciable inflammatory responses [68]. Modeling adenovirus infection in vitro with HAdV-5-infected HiPSC-CMs or in vivo with MAdV-3-infected mice demonstrated adenoviral infection resulted in hyperphosphorylation of Cx43-S368, decreasing channel opening probability and gap junction function [59–61, 68]. Adenovirus infection-induced Cx43-S368 hyperphosphorylation was confirmed to depend upon PKC activation, as pharmacological inhibition of PKC during HAdV-5 infection abolished Cx43-S368 hyperphosphorylation [68]. Moreover, Cx43-S368 hyperphosphorylation is causative to an arrhythmogenic substrate in MAdV-3 infection: mice harboring a phospho-null mutation in Cx43-S368 (Cx43-S368A mice) infected with MAdV-3 were protected against conduction velocity slowing observed in infected wild-type animals [68]. Corroborating in vivo findings and further elucidating mechanisms of adenoviral-mediated gap junction perturbation, in vitro models of adenoviral infection demonstrate HAdV-5 also significantly decreases Cx43 mRNA and protein levels through activating β-catenin [69]. Prior to decreasing Cx43 expression, HAdV-5, just as observed with MAdV-3, inhibits gap junction function through inducing hyperphosphorylation of Cx43 at S373 and S368, while decreasing interaction between Cx43 and the scaffolding protein zonula occludens-1 (ZO-1) [6, 59–61, 69, 70]. One possible mechanism for adenoviral-mediated perturbation of Cx43 may involve viral proteins encoded within the E4 genome region, which have been reported as sufficient to decrease endothelial and cardiac expression of Cx43 through activation of protein kinase A (PKA) and PI3K [71].

While these studies demonstrate a direct viral-mediated regulation of Cx43, another potential mechanism through which adenoviral infection may modulate connexin expression is through interactions with its host cell receptor, the coxsackie and adenovirus receptor (CAR) [72, 73]. Loss of cardiac CAR expression is associated with decreased Cx43 expression, resulting in impaired gap junction function and aberrant cardiac electrophysiological properties [73]. As the fiber protein of adenovirus interacts with CAR to induce paracellular permeability promoting viral release, this suggests that adenoviral modulation of CAR expression and/or localization may also contribute to these observed decreases in Cx43 expression underlying arrhythmias observed in acute viral myocarditis [74]. In addition, fiber-mediated disruption of adhesion at the ID would precipitate additional arrhythmogenic effects through disruption of the complex arrangement of nanodomains and junctions involved in normal cardiac electrical coupling and contractility [75, 76]. Together, these studies indicate adenoviral infection leads to subversion of gap junction function in a multipronged manner, as adenovirus decreases Cx43 expression, induces PTMs altering gap junction function, and disrupts interactions between Cx43 and its binding partners.

#### Coxsackievirus B3

Enteroviruses, including the CVB3, are another leading etiological cause of viral myocarditis, with viral RNA detected in myocardial biopsies from viral myocarditis patients [62]. Mouse models of CVB3-induced myocarditis demonstrate CVB3 infection decreases cardiac Cx43 and Cx45 expression and results in lateralization of Cx43 [48, 77, 78]. Despite the established relationship between CVB3 infection and cardiac connexin expression, our current understanding of CVB3-induced connexin remodeling remains limited and focuses primarily on microRNA (miRNA)-mediated posttranscriptional regulation. CVB3 infection increases cardiac expression of the microRNAs miR-1 and miR-19b; exogenous expression of either miRNA sufficient to decrease Cx43 expression in vitro [77, 78]. Interplay may exist between miR-1 and miR-19b, as co-expression synergistically decreases Cx43 expression [78]. Overall, this suggests that increased miR-1 and miR-19b expression in CVB3-induced viral myocarditis may contribute to the observed decrease in cardiac Cx43 expression.

In addition to its ability to directly perturb connexin dynamics, CVB3 may also indirectly induce changes in connexin dynamics through costameric/sarcolemma complex disruption. CVB3 protease 2A directly cleaves dystrophin in cardiomyocytes [79, 80]. Dystrophin cleavage during CVB3 infection has been demonstrated to disrupt the sarcolemma and lead to worsened myocarditis severity [81]. CVB3-mediated cleavage of dystrophin, which drives altered sarcolemmal structure, may contribute to altered Cx43 dynamics: in cardiac tissue from Duchenne muscular dystrophy (DMD) patients and mouse models, both lacking functional dystrophin, increased Cx43 expression and lateralization away from the ID have been observed [82]. Together, this suggests that CVB3-mediated cleavage of dystrophin may also underlie perturbations in cardiac connexin dynamics.

#### Human Immunodeficiency Virus

Another virus that both directly and indirectly contributes to viral myocarditis and other cardiovascular complications is human immunodeficiency virus (HIV) [83]. Viral genetic material has been detected in cardiac tissue obtained from HIV+ patients, suggesting direct HIV infection of the heart [84, 85]. As HIV infection increases the incidence of cardiovascular complications, this suggests a relationship between HIV infection and cardiac connexin expression and function. Interestingly, Cx43 mRNA and protein levels are increased in cardiac tissue from HIV+ individuals, and Cx43 lateralization was also observed in cardiomyocytes [22]. Moreover, ventricular regions with increased Cx43 expression and lateralization also had increased Ca^2+^ levels, fibrosis, and an increased number of mitochondria, indicating a potential link between Cx43 and cardiac damage in the context of HIV infection [22].

While an understanding of how HIV perturbs cardiac connexins has yet to be fully characterized, our current understanding of HIV-mediated subversion of connexins within the central nervous system (CNS) may provide some mechanistic insight. Similar to observations within cardiac tissue, HIV has been demonstrated to increase Cx43 expression in various cell types of the CNS, including astrocytes, pericytes, and neural progenitor cells [86–88]. This HIV-induced increase in Cx43 expression is associated with increases in gap junction function and hemichannel activity [87–89]. Interestingly, gap junction function has been identified as a key contributor to HIV-mediated bystander cell death and viral infection, as inhibition of Cx43 gap junctions decreases bystander cell apoptosis and progression of viral infection [86, 87, 90, 91]. Bystander cell death has been attributed to gap junction-mediated spread of inflammatory mediators, including CCL2, and secondary messengers that lead to an increase in intracellular Ca^2+^, such as a cytochrome C-induced increase in inositol triphosphate [86, 90]. Investigation of viral factors sufficient to perturb Cx43 expression and function has identified the HIV trans-activator of transcription (tat) as a key factor driving increased astrocytic Cx43 mRNA and protein levels, resulting in increased gap junction function [92]. HIV-tat binds directly to the *GJA1* promoter, suggesting HIV-tat may increase Cx43 expression through activating *GJA1* transcription [92]. This is specific to the *GJA1* promoter: despite its ability to decrease expression of Cx30, binding of HIV-tat to the *GJB6* promoter was not observed [92].

The ability of HIV to manipulate connexins has also been investigated within the context of macrophage-derived tunneling nanotubules (TNTs). HIV infection increases macrophage expression of Cx43, which localizes to the base and tip of the TNT [91]. Long distance cell–cell communication was facilitated by TNT-localized Cx43, as pharmacological inhibition of gap junctions abolished dye transfer between TNT-adjoined macrophages [91]. Interestingly, inhibiting either TNT formation or gap junction function diminished HIV infection and replication; however, the presence of viral particles was not detected within the TNTs, suggesting gap junctions within TNTs facilitate the spread of signaling molecules that predispose uninfected macrophages to HIV infection [91].

Collectively, research investigating HIV-mediated regulation of connexins demonstrates that HIV infection increases Cx43 expression, along with hemichannel activity and gap junction function. Ultimately, this facilitates viral propagation and the release of cytotoxic mediators, resulting in viral spread and bystander cell death. As increased Cx43 expression is also observed in cardiac tissue from HIV+ patients, this suggests the potential of a conserved mechanism of HIV-induced cell damage across tissue types. For example, increased cardiac Cx43 expression in cardiomyocytes may result in increased gap junction function and hemichannel activity, leading to bystander cell damage which could underlie the cardiovascular complications observed in HIV+ patients.

#### Influenza Virus A

Although less common, influenza A virus (IAV) has also been identified as an etiological agent of viral myocarditis [62]. Human IAV has been demonstrated to directly infect both mouse cardiomyocytes and Purkinje cells in vivo [93]. The infection of the latter may underlie the observed changes in cardiac conduction, with electrocardiography demonstrating significantly longer PR intervals, RR intervals, and QRS duration in IAV-infected mice compared to control mice [93]. In human induced pluripotent stem cell-derived cardiomyocytes (HiPSC-CMs), IAV infection resulted in an acute decrease in protein levels of Cx43 and the sodium channel Na_V_1.5, which also may contribute to the altered cardiac electrophysiological properties observed upon IAV infection [93]. As the relationship between IAV infection and viral myocarditis is well-established, understanding IAV-mediated perturbations to connexins expressed in the working myocardium and the cardiac conduction system will be important in understanding cellular alterations driving IAV-induced myocarditis.

### Other Viruses Underlying Cardiac Complications

#### Zika Virus

While rare, case studies of myocarditis and other cardiovascular complications have been reported in individuals infected with Zika virus (ZIKV) [94–96]. Use of non-human primates and mice to model ZIKV infection demonstrates detectable ZIKV within cardiac tissue [97–99]. Likewise, immunostaining of cardiac tissue from ZIKV-infected mice reveals ZIKV protein localization within cardiomyocytes, and ZIKV can infect HiPSC-CMs [98, 99]. Unsurprisingly, ZIKV-infected mice display cardiac abnormalities, including increased serum levels of acute myocardial injury biomarkers, fragmentation and prolongation of the QRS complex, prolonged PR duration, and altered ID structure [99]. Despite this emerging understanding of ZIKV infection and cardiovascular disease, much remains to be clarified regarding the relationship between ZIKV infection and connexin regulation; however, ZIKV has been demonstrated to downregulate Cx43 expression via proteasomal degradation [99].

#### Coronaviruses

Infection with severe acute respiratory syndrome coronavirus 2 (SARS-CoV-2) is associated with an increased incidence of cardiovascular complications, including myocardial injury, myocarditis, and arrhythmias [100–102]. Coronavirus disease 19 (COVID-19) has also been identified as an independent risk factor for myocardial infarction [103]. Current evidence suggests SARS-CoV-2 may be both directly and indirectly involved in viral myocarditis: while SARS-CoV-2 viral RNA has been detected in cardiac tissue in a subset of patients with myocarditis and heart failure, localization of SARS-CoV-2 viral proteins within cardiomyocytes is sparse, with viral proteins primarily localizing to cardiac endothelial cells [104, 105]. With the increased prevalence of cardiovascular complications following SARS-CoV-2 infection, understanding how SARS-CoV-2 affects cardiac Cx43 dynamics is of high relevance to the field. Our understanding of SARS-CoV-2-mediated Cx43 subversion, let alone cardiac Cx43 subversion, is nascent. Interestingly, SARS-CoV-2 spike protein treatment induces re-localization of Cx43 from the cell periphery to the nuclear and perinuclear regions in HiPSC-CMs [106]. In Cx43-expressing HeLa cells, the S1 subunit of the SARS-CoV-2 spike protein activates Cx43 hemichannels while impairing GJIC, resulting in increased ATP release and altered intracellular Ca^2+^ dynamics [107]. Ultimately, this suggests Cx43 hemichannel activation may underlie cellular damage within the context of SARS-CoV-2 infection [107]. As spike S1 has been demonstrated to increase intracellular Ca^2+^ levels, a known trigger for Cx43 hemichannel activity, the authors speculate that this may be a potential mechanism through which spike S1 leads to Cx43 hemichannel activation [107–110].

Because our current understanding of how SARS-CoV-2 infection perturbs connexins is limited, research investigating how other betacoronaviruses alter connexin expression and function may provide mechanistic insight. For example, human coronavirus-OC43 (HCoV-OC43) infection decreases expression of Cx43 and results in its perinuclear localization, functionally translating to an impairment in both hemichannel activity and gap junction function [111]. This perturbation of Cx43 dynamics may occur through viral-induced ER stress, as HCoV-OC43 infection increases expression of ER stress markers and disrupts the Golgi apparatus [111]. Aside from HCoV-OC43, extensive research has been conducted to characterize the effects of murine hepatitis virus (MHV) infection on Cx43 regulation. MHV-A59, a neurotropic strain of MHV, has been demonstrated to decrease Cx43 expression in vivo and in vitro across CNS cell types, including primary mouse astrocytes and meningeal fibroblasts [112, 113]. Additionally, MHV-A59 concomitantly decreases Cx47 expression in vivo [114]. Similar to HCoV-OC43, MHV-A59 infection results in re-localization of Cx43 from the cell periphery to the perinuclear region [112, 113, 115]. Perinuclear localization of Cx43 may be due to viral-induced disruption of Cx43 and β-tubulin interactions or decreased expression of ERp29, an ER Cx43 chaperone [114, 115]. With MHV-A59 infection influencing both Cx43 expression and localization, it is unsurprising that MHV-A59 decreases gap junction function [112, 113]. Ultimately, decreased gap junction function aids in viral progression, as inhibiting gap junction function during MHV-A59 infection further increases viral infectivity, while rescuing gap junction function decreases viral progression [115]. As HCoV-OC43 and MHV-A59 both disrupt expression and localization of Cx43, this suggests that altered Cx43 dynamics may be conserved across coronaviral infections, including SARS-CoV-2 infection.

## Potential Therapeutic Strategies

While current treatments for cardiac viral infection and myocarditis remain largely supportive, targeting specific arms of the immune response to tackle inflammation is progressing at the clinical level. Immuno-modulatory approaches also encompass limiting auto-immunity, and in some cases specific-antiviral therapy may be available [116]. One anti-inflammatory approach that has gained traction involves inhibition of IL-1, as reviewed in [117]. Specifically, treatment with the IL-1 receptor antagonist (IL-1ra) anakinra has been demonstrated to resolve impaired cardiac function in both adult and pediatric myocarditis patients [118–120]. These experimental findings served as the rationale for the ARAMIS trial (ClinicalTrials.gov identifier: NCT03018834), a phase II randomized double-blind trial testing the efficacy of anakinra for acute myocarditis treatment [121]. The ARAMIS trial, however, did not demonstrate a difference in outcomes between anakinra- and placebo-treated patients, which may be due to the small sample size and the inclusion of low-risk patients with various etiological causes of acute myocarditis [122]. Potentially, future clinical trials with larger sample sizes and more stringent inclusion/exclusion criteria may be needed to fully explore the potential of anakinra as a myocarditis treatment [122]. As specific viral mechanisms of cardiac insult are actively parsed out, future strategies for treating viral myocarditis may involve targeting virally-activated signaling pathways that perturb connexin function, such as PKC signaling [68, 69]. Between the ubiquitous expression of PKC isozymes likely leading to off-target effects and the lack of selective PKC inhibitors, however, inhibiting PKC to treat viral myocarditis may prove challenging [123]. Ultimately, this underscores the importance in delineating the direct and indirect contributions to cardiac connexin and electrical coupling perturbation during viral infection.

## Conclusions

Current research indicates connexins as a common viral target, as alterations in connexin expression, localization, and function are observed across various viral infections, including the known etiological agents of viral myocarditis (summarized in Table 1). Despite an established relationship between viral infection and connexin perturbation, this has only recently become an active area of investigation within the context of viral myocarditis. As such, future research is needed to fully delineate the mechanisms underlying viral-mediated subversion of cardiac connexins. The development of physiologically relevant in vitro models, including HiPSC-CMs, and in vivo models, such as mouse-specific/adapted viral strains, is already enabling relevant elucidation of molecular mechanisms contributing to alterations in connexin expression and function during viral infection. Much of the current research investigating viral-mediated alterations to connexins has focused on Cx43; however, expanding our understanding of viral-mediated regulation of other cardiac connexins will be important to fully understand pathological changes to conduction occurring during viral myocarditis. Furthermore, a comprehensive understanding of both direct (viral-mediated) and indirect (inflammation-mediated) contributions to connexin subversion will need to be elucidated, as understanding mechanism(s) will aid in the identification of novel therapeutic targets for viral myocarditis. This is especially important given the phase of infection and etiological agent of viral myocarditis, as inflammation and infiltrating immune cells predominate during the later stages of disease and are common across etiological agents [124]. Ultimately, identifying mechanisms of cardiac connexin perturbation will aid in the identification of novel substrates for therapeutic treatment of viral myocarditis, preventing sudden cardiac death in acute cases and the progressive chronic path to heart failure.

**Table 1:** Summary of viral-mediated alterations to connexins

Category	Virus	Alterations to connexins
Adenoviruses	HAdV-5	Decreased Cx43 expression[69] Decreased GJ function[69] Disrupted Cx43/ZO-1 interactions[69] Cx43-S373 hyperphosphorylation[69] Cx43-S368 hyperphosphorylation[68, 69]
	MAdV-3	Cx43-S368 hyperphosphorylation[68]
Enteroviruses	CVB3	Decreased Cx43 expression[48, 77, 78] Decreased Cx45 expression[48] Lateralization and/or reduced ID Cx43[48, 77]
Lentiviruses	HIV	Increased Cx43 expression[22, 86–88] Cx43 expression within macrophage TnTs[91] Cx43 lateralization in cardiomyocytes[22] Increased GJ function[87, 88] Increased hemichannel activity[87–89] HIV-Tat increases Cx43 expression and GJ function[92]
Influenzaviruses	IAV	Decreased Cx43 expression[93]
Flaviviruses	ZIKV	Decreased Cx43 expression[99]
Coronaviruses	SARS-CoV-2	Spike protein results in nuclear and perinuclear Cx43 localization[106] S1 spike protein subunit activates Cx43 hemichannels and impairs GJIC[107]
	HCoV-OC43	Decreased Cx43 expression, perinuclear localization, and impaired HC and GJ activity[111]
	MHV-A59	Decreased Cx43 expression[112, 113] Decreased Cx47 expression[114] Perinuclear localization of Cx43[112, 113, 115] Disrupted Cx43/β-tubulin interactions[114] Decreased GJ function[112, 113]

Proper connexin expression and localization are crucial for rapid action potential propagation and the coordinated contraction of the heart; aberrancies in connexin regulation occur in many cardiovascular disease states and underlie arrhythmogenesis. Connexins also possess a unique immunomodulatory role: gap junction function is crucial for the propagation of immune signaling molecules; however, hemichannel activity can promote the spread of cytotoxic signals and result in cell damage or death. As such, it is unsurprising that connexins are commonly targeted by viruses in order to promote viral propagation and that this targeting of connexins is observed in viral myocarditis. Research investigating viral myocarditis and viral infection highlights connexin perturbation, whether directly or indirectly mediated by viral infection, as a common mechanism underlying pathology. This subversion of connexin function ultimately facilitates viral propagation and cell damage, contributing to the progression of viral myocarditis. As such, understanding mechanisms of connexin perturbation during viral myocarditis is crucial, providing new antiviral targets for therapeutics to prevent progression of viral myocarditis.

## Key References

- Padget RL, Zeitz MJ, Blair GA, Wu X, North MD, Tanenbaum MT, Stanley KE, Phillips CM, King DR, Lamouille S, Gourdie RG, Hoeker GS, Swanger SA, Poelzing S, Smyth JW. Acute Adenoviral Infection Elicits an Arrhythmogenic Substrate Prior to Myocarditis. Circulation research. 2024;134(7):892–912. Epub 20,240,228. doi: 10.1161/CIRCRESAHA.122.322437. PubMed PMID: 38,415,360; PMCID: PMC11003857.
  - Identified acute viral infection induces arrhythmogenic alterations to connexin function prior to appreciable myocarditis or cardiomyopathy/damage.
- Karmakar S, Das Sarma J. Human coronavirus OC43 infection remodels connexin 43-mediated gap junction intercellular communication in vitro. J Virol. 2024:e0047824. Epub 20,240,531. doi: 10.1128/jvi.00478-24. PubMed PMID: 38,819,132.
  - Of relevance to COVID-19, demonstrates gap junction function is affected during human coronavirus infection.

## Data Availability

No datasets were generated or analysed during the current study.
